# Flexible and efficient genome tiling design with penalized uniqueness score

**DOI:** 10.1186/1471-2105-13-323

**Published:** 2012-12-05

**Authors:** Yang Du, Eduard Murani, Siriluck Ponsuksili, Klaus Wimmers

**Affiliations:** 1 Research Unit Molecular Biology, Leibniz Institute for Farm Animal Biology, Dummerstorf, Germany; 2 Research Group Functional Genomics, Leibniz Institute for Farm Animal Biology, Dummerstorf, Germany

**Keywords:** Tiling array, Targeted sequencing, Probe design, Penalized uniqueness score

## Abstract

**Background:**

As a powerful tool in whole genome analysis, tiling array has been widely used in the answering of many genomic questions. Now it could also serve as a capture device for the library preparation in the popular high throughput sequencing experiments. Thus, a flexible and efficient tiling array design approach is still needed and could assist in various types and scales of transcriptomic experiment.

**Results:**

In this paper, we address issues and challenges in designing probes suitable for tiling array applications and targeted sequencing. In particular, we define the penalized uniqueness score, which serves as a controlling criterion to eliminate potential cross-hybridization, and a flexible tiling array design pipeline. Unlike BLAST or simple suffix array based methods, computing and using our uniqueness measurement can be more efficient for large scale design and require less memory. The parameters provided could assist in various types of genomic tiling task. In addition, using both commercial array data and experiment data we show, unlike previously claimed, that palindromic sequence exhibiting relatively lower uniqueness.

**Conclusions:**

Our proposed penalized uniqueness score could serve as a better indicator for cross hybridization with higher sensitivity and specificity, giving more control of expected array quality. The flexible tiling design algorithm incorporating the penalized uniqueness score was shown to give higher coverage and resolution. The package to calculate the penalized uniqueness score and the described probe selection algorithm are implemented as a Perl program, which is freely available at http://www1.fbn-dummerstorf.de/en/forschung/fbs/fb3/paper/2012-yang-1/OTAD.v1.1.tar.gz.

## Background

As a unique variety of microarray, genome tiling array targets not only known transcripts that are dispersed across the genome, but intensively covers all known contiguous regions on the genome with overlapping or evenly-spaced probes. Being more unbiased than common gene expression arrays, tiling arrays have the ability to empirically investigate transcriptional activity across all known genomic regions, some of which may contain unknown transcripts and splicing variants. Studies using tiling arrays in human and Arabidopsis thaliana have shown that the number of transcripts exceeds predictions by up to 10 times, and further, many exist outside of annotated exons [1,2]. Besides transcriptome mapping, other applications also make tiling array an ideal tool for whole-genome analysis [3,4], including ChIP-on-chip for DNA-protein interactions, MeDIP-chip for DNA methylation, DNase chip for identification of hypersensitive sites, and array comparative genomic hybridization (aCGH) to study DNA copy number variation.

However, in recent years with advancing technology and lower costs, high-throughput sequencing (next generation sequencing, NGS) has gained unprecedented attention in genome research. With its inherent single-base resolution, NGS achieves higher accuracy in exon boundary mapping and can be used for SNP detection [5]. Further, its unlimited dynamic range enables detection of any subtle changes in the transcription level [6]. Despite significant cost reduction for NGS, tiling array remains more cost-effective for large samples — which typically provide higher and more reliable statistical power — therefore, cross-platform collaboration between deep sequencing data and array data has been considered [7].

Additionally, relatively few ongoing research projects seek to solve biological questions on a whole-genome scale, so it is more likely that several linked QTL regions or intervals identified in genome-wide association studies are pursued in detail. Such specific interests can be addressed by using capture oligos for targeted enrichment of DNA fragments representing the genomic region of interest [8]. These capture oligos might be applied either in solution-based or microarray-based methods, thus combining the two platforms in the array assisted targeted sequencingapproaches, where targeted regions could be tiled on DNA capture arrays, and the hybridization products could be used in the follow-up sequencing library preparation [9-11] This customized tailoring tool selectively enriches only the regions of interest and provides the opportunity to reduce both cost and processing time, while retaining high sensitivity through high-throughput technology.

In this paper, we would like to address issues and challenges in designing probes suitable for genome tiling and targeted sequencing. In particular, we present the penalized uniqueness score, which serves as a controlling criterion to eliminate potential cross-hybridization, and a flexible tiling array design pipeline.

## Results

### Penalized uniqueness score

In microarray design, selected probes should have similar hybridization efficiencies under a specified narrow band of temperature, as well as minimal potential for both self-hybridization and cross-hybridization [12]. However, these selection constraints become much more difficult to satisfy when applied to tiling probes designed for a large genomic region. Among these conditions, cross-hybridization is most problematic, when a non-targeted nucleotide sequence hybridizes to the designed probe. Cross-hybridization contributes to the overall probe signal intensity and may potentially compromise the downstream analysis [13]. A similar situation with lower specificity also troubles deep sequencing, when short reads need to be either aligned to a reference genome or assembled into contigs [14].

Experimental techniques and analytical methods have been developed to overcome this issue. Commonly, approaches to probe design are mostly BLAST [15] based [16-20]; other researchers [21-23] also employ suffix array [24] for faster indexing and alignment, however at the cost of increasing space complexity.

Furthermore, Gräf *et al.*[25] proposed the idea of uniqueness score (*U*) for cross-hybridization control in tiling probe selection. Following their original definition, a genome sequence in question is called G, which is a part of the whole genome assembly GS. If a substring X of G occurs only once in GS, and each substring of X occurs more than once in GS, then this substring X is called a minimum unique substring (MUS) of G. At each position of G, if the substring from this position to the end of G is unique within GS, then there exists one shortest unique prefix starting from that position, which is called a minimal unique prefix (MUP) at that position. The uniqueness score is then defined as the number of MUS within any candidate genome interval; finding *U* could then be achieved by counting the distinct end positions of MUP within the region. Details and proof could be found in [25].

**Figure 1:**
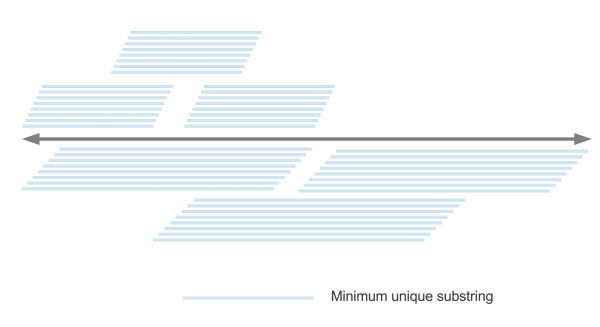
**Schematic diagram of low MUS coverage and long MUS.** Hypothetically extreme cases showing low MUS coverage (above) and long MUS (below).

However, in this definition of the score, they only considered the absolute number of MUS without accounting for the distribution of length and coverage within the probe. Hypothetically, the first half of a probe could contain no MUS, while the remainder might harbor a large number of MUS (which could be up to the user-specified cutoff). In certain extreme cases, for longer oligonucleotide probes, the original definition would give a score higher than the specified threshold, even though the actual coverage of these MUS is only 50%, and cross-hybridization could still occur. Another possible scenario is that compared to sequences with shorter MUS, probes with longer and near-window-sized MUS are more vulnerable to cross hybridization. A schematic illustration of the 2 aforementioned cases is shown in Figure 1; some potentially vulnerable probes from one typical array can be found in Table 1.

**Table 1:** Example of 60-mer probes from Agilent ChIP-on-Chip Set

**Probe ID**	**BLAT hits**	**MUS coverage**	**MUS Avg. Len.**	***U***	***U***_***p***_
A_17_P01761220	2	45	17.55	22	6.85
A_17_P17428106	2	44	16.42	24	7.97
A_17_P04386305	2	59	20.86	22	6.59
A_17_P10898621	12	55	20.86	22	6.14

BLAT hits, number of hybridization-quality alignment to the reference genome. MUS coverage is the length of the region on the probe covered by MUS. MUS Avg. Len, is the average length of all MUS within the probe. *U*, the original uniqueness score. *U*_*p*_, the penalized uniqueness score.

Hence we define the penalized uniqueness score *U*_*p*_ as the following,

(1) $$U_{p}=U\times C_{\mathrm{mus}}\times \left(1-mean\left(L_{\mathrm{mus}}\right)/L_{\max}\right)$$

*C*_*mus*_, is the proportion of the probe covered by all MUS within.

*L*_*mus*_, is length of the MUS within the candidate probe.

*L*_*max*_, is the maximal length of MUP, default is 30.

*U*, is the number of MUS within the candidate probe.

Distribution quartiles of model parameters for one Agilent chip are shown in Table 2. So in the best case scenario, all MUS cover the whole probe and the coverage will induce no penalty on the uniqueness score. Regarding the average length of MUS, it will normally give a coefficient between 0 (only if all MUS within the probe have maximal length) and 1 (only when there is no MUS available).

**Table 2:** Summary of MUS coverage ratio and average length (in bp) of Agilent ChIP-on-Chip Set probes

	**Min.**	**1st Qu.**	**Median**	**Mean**	**3rd Qu.**	**Max.**
*C*_*mu*s_	0.23	0.96	0.97	0.97	0.98	1
*mean(L*_*mus*_*)*	13.26	16.91	17.32	17.33	17.76	30

### Penalized uniqueness score evaluation

**Figure 2:**
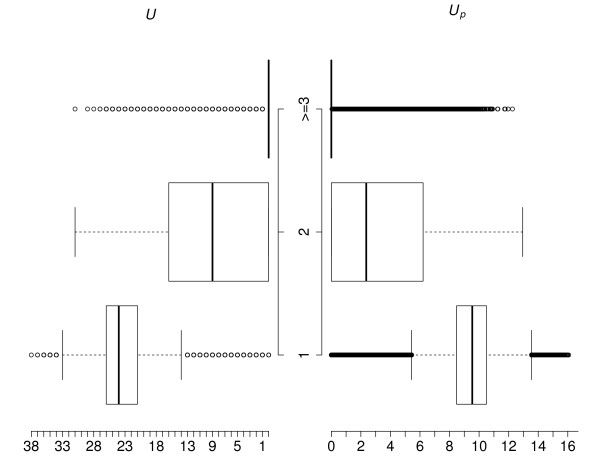
**Back-to-back box-plot.** Back-to-back box-plot of original uniqueness score (*U*, left) and the penalized uniqueness score (*U*_*p*_, right) distribution within different BLAT hits groups (1, aligned to 1 position; 2, aligned to 2 positions; ≥3, aligned to at least 3 positions).

After BLAT [26] alignment, 99.4% of all Agilent Human Whole Genome ChIP-on-Chip probes found to have unique quality alignment, while only 34274 probes had been mapped to multiple locations. We made back to back box-plots (Figure 2) for both scores to visualize the general group differences in their distributions. Both plots show similar overall pattern. Probes with unique alignment tend to have a higher score. A visible difference of the grouping effect between the two scores could be found, that for the penalized uniqueness score the difference of median between group 2 and 3 is much lower than the difference of median between group 2 and group 1. An estimate of grouping effect, calculated using below formula (2), would give *U*_*p*_ a ratio of 3.03557, almost two times higher than 1.66667 for *U*, suggesting a larger difference in the distribution of the penalized score between unique probe group and probes with multiple alignments.

(2) $$\frac{median\left(U_{1}\right)-median\left(U_{2}\right)}{median\left(U_{2}\right)-median\left(U_{\geq 3}\right)}$$

**Figure 3:**
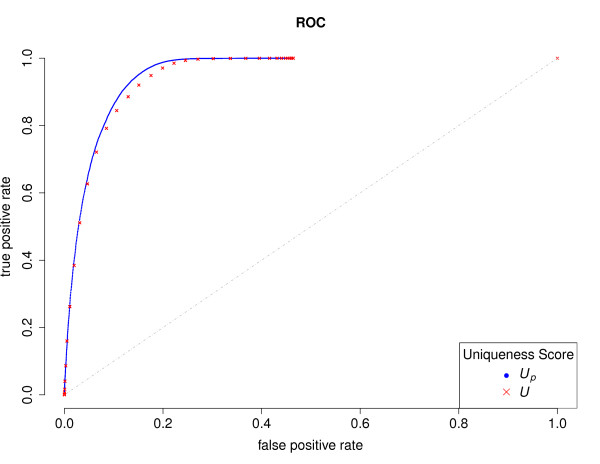
**Receiver operating characteristic curve.** ROC curves of using original uniqueness score (*U*_*p*_, red) and penalized uniqueness score (*U*, blue) for BLAT hits group classification.

To access directly the relative performance of the two scores, receiver operating characteristic curve (ROC) was adopted measuring the trade-off between sensitivity and specificity. Curves for both score were overlaid on Figure 3. It could be seen that both scores work quite well and strongly deviate from the diagonal, however, a visible trace of difference could be identified at the upper left corner, suggesting a clear gain of advantage by the penalized score.

### Design parameters

The melting temperature (Tm), defined as the temperature at which 50% of the oligonucleotide and its perfect complement are in duplex and the other half are in the random coil state, is essential for the success of stable hybridization, where a narrow band of Tm across all probes is highly desirable [27]. Common thermodynamics prediction models utilizing only base composition, like GC content [28] or base counts [29], have been proposed and widely used for short oligonucleotides. However, these simple models do not consider the actual probe sequence, but rather use only the summary statistics of the sequence. The loss of information will inevitably lead to lower prediction power. The nearest-neighbor model later proposed is considered more robust and accurate [30]. Thus, in this algorithm we implemented the adapted prediction model of Tm (3) using the same parameters as in [31]. Aside from Tm, the GC content could also be controlled separately.

(3) $$\begin{array}{c} Tm=\frac{\sum \left(\Delta H_{d}\right)+\Delta H_{i}}{\sum \left(\Delta S_{d}\right)+\Delta S_{i}+\Delta S_{\mathrm{self}}+R\times lnC_{T}/b}\\ \;+16.6\times \log\left[Na^{+}\right] \end{array}$$

**Figure 4:**
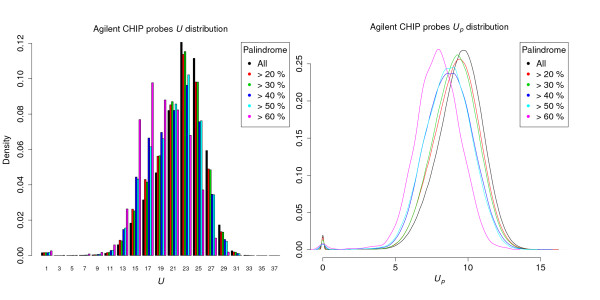
**Palindromic content and uniqueness scores of Agilent ChIP-on-Chip Set.** Distributions of the original uniqueness score (*U*, left) and penalized uniqueness score (*U*_*p*_, right) of the Agilent Human Whole Genome ChIP-on-Chip Set 244K (1 to25) probes, using different level of palindromic content as cutoff.

To limit self-hybridization potential, palindromic content, measured as the maximal proportion of inverted repeat (IR), could be controlled via a user-specified threshold. Interestingly, unlike in [25], where the author claimed that palindromic sequences are more unique in the genome, we observed an opposing relationship between palindromes and uniqueness (in Figure 4): Agilent's catalog probes with higher palindromic content tended to have lower mean uniqueness scores, which caused a left-shifting of their distributions when using a high palindrome cutoff. Small yet significant correlations could also be detected for both uniqueness scores *U*, -0.0736428 (p<2.2e-16); *U*_*p*_, -0.1050423 (p<2.2e-16)] and BLAT hits [0.00209 (p=3.578e-07)] with palindromic content. However, the distribution of BLAT scores is extremely left-tilted, since most probes are aligned only once, therefore, despite supporting our findings, the correlation test may not be valid.

Repetitive regions, as one major source of cross-hybridization, account for a large proportion of mammalian genomes like human and pig. The most commonly adopted approach to handle repetitive regions is to exclude those using tools like RepeatMasker (http://repeatmasker.org) or Window Masker [32]. However, evidence suggests a role for repetitive DNA in gene conversion; therefore not all repeats are “junk” [33]. Tandem repeats have also been shown to be associated with regulation of transcription factor binding [34,35] and various disease forms including cancer [36,37]. Thus, when the uniqueness score is used to control cross-hybridization, regions, that were masked as repetitive but had a high uniqueness score, could still be included. Therefore it is suggested that, as an optional but recommended preprocessing step, target regions of the genome could be masked with the aforementioned tools. Further, a parameter controlling the proportion of repetitive masked regions on the candidate probe is available, allowing for either full exclusion of repetitive regions or partial inclusion of a specified proportion. Back-mapping Agilent chip targets to the reference sequence also suggests inclusion of repetitive sequence, with 411292 probes having a repetitive proportion greater than zero, and 199471 probes being completely masked as repetitive.

### Probe selection algorithm

Tiling arrays can be designed in the most straightforward way, either using an end-to-end fashion or with a fixed distance or overlap between neighboring tiles. However, these simple strategies will easily encounter problems like cross-hybridization and low hybridization potential, problems that would eventually contaminate the data. Also, instead of having a high coverage up to 100% initially, the number of probes with valid signals could fall significantly in the end. To gain more control of the expected quality while giving better resolution and coverage, we present the following design strategy and pipeline.

The algorithm searches for candidate probes in an intuitive growing fashion, from the 5' of the sequence to the 3' end. In general, we attempted to make neighboring probes having a fixed size of overlap, if the targeted probe satisfies the user-specified constraints. Otherwise, the adjacent positions overlapping with 1 nt more or less would be tested, and the search would keep shifting until the next valid probe is found or the boundary of the genomic region is reached. Pseudo code of the detailed selection mechanism is shown in the algorithm (section “Pseudo code of the probe selection algorithm”. An evenly-spaced, non-overlapping tiling path can be achieved by specifying a negative value for the overlap size. Another separate option of flexible probe length can be combined with the overlapping option to compensate for coverage in regions where fixed-length probes cannot be placed. Strand-specific design is also possible; if both strands are present on the same array, offsets between reverse compliment tiles on the two strands are determined internally.

Pseudo code of the probe selection algorithm

start from the 5’

while still enough space to place a probe

if probe temperature is too high

if all position to the 5' has been checked

jump to last checked position to 3’, shift 1 nt to 3' and re-check

else if probe length can be shorter

probe length minus 1 and re-check

else if it is ok to shift to 5’

shift 1 nt to 5' and re-check

else if probe temperature is too low or not unique enough

if all possible positions to the 5' has been checked

jump to last checked position to 3’, shift 1 nt to 3' and re-check

else if probe length can be shorter and ok to shift to 5’

probe length plus 1, shift 1 nt to 5' and re-check

else if it is ok to shift to 5’

shift 1 nt to 5' and re-check

if other remaining checks failed

if all position to the 5' has been checked

jump to last checked position to 3’, shift 1 nt to 3' and re-check

else if probe length can be shorter and ok to shift to 5’

probe length plus 1, shift 1 nt to 5' and re-check

else if it is ok to shift to 5’

shift 1 nt to 5' and re-check

tile found

move to next candidate position

end loop

### Design comparison

For the Agilent catalog array set, we evaluated all the probes based on our design parameters, figures of all parameters' distribution could be found in the Additional file 1. In general, the catalog probes show similar hybridization efficiency, with an inter quartile range of melting temperature from 67.98 to 73.08, low cross hybridization potential, with an average uniqueness score of 23.06 (median 24) and penalized uniqueness score of 9.412 (median 9.53), and limited self-hybridization potential, having a mean palindromic content of 22.39% (median 20%). To address the coverage and resolution of our design, we chose the whole human chromosome 22 (GenBank:NC_000022.10) as the target region, and detail settings could be found in method.

With our implementation, it took around 2 hours on a 3.0 Ghz single core PC to finish the process. In Table 3, we summarize the Agilent catalog design and our custom designs, showing that our flexible strategy could achieve higher coverage with fewer and, on average, longer probes. The coverage was assessed using two types of measurement, the raw non-redundant bases covered (ambiguous base 'N' adjusted) and the total length of unit-sized (1000 bp) windows in which at least one probe was placed.

**Table 3:** Design summary and coverage comparison

	**Probe no.**	**Probe length**^**‡**^	**Inter probe distance**^**‡**^	**Base coverage**	**Window coverage**
Agilent*	73373	53/52.85	202/426	11.11%	51.25%
-o −250^†^	75036	60/57.16	246/411.9	12.29%	53.60%
-o −275	72087	60/57.11	269/431.1	11.80%	53.66%
-o −300	69491	60/57.02	292/449.4	11.35%	53.74%
-o −325	67074	60/56.92	313/467.7	10.94%	53.81%

* Agilent Human Whole Genome ChIP-on-Chip Set 244K (Chromosome 22 only).

† Overlapping size set to −250, probe length range [45, 60], Tm range [69, 74], *U*_*p*_ range [9, Inf), palindromic content range [0, 30%], GC content range [30%, 50%].

‡ Table cell is formatted as median/mean.

¶ Using the length of all unit-sized (1000 bp) windows in which at least one probe was placed.

### Uniqueness of palindromic sequence

To investigate further the relationship between sequence uniqueness and palindromic content, we utilized data from a separate experiment in which we used our implementation to design tiling arrays for several chromosome regions of the pig genome. For one of them, on *Sus Scrofa* chromosome 14 (GenBank:NC_010456.3) from 74377028 bp to 78176022 bp, we built the profile of all design parameters for all possible 60-mer probes in the indicated region. In total 3328358 candidates were evaluated.

**Figure 5:**
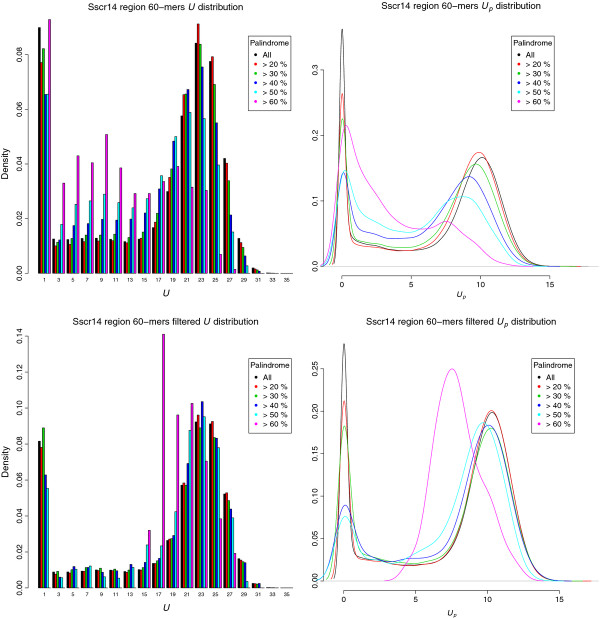
**Palindromic content and uniqueness scores of experimental data.** Distributions of the original uniqueness score (upper-left) and penalized uniqueness score (upper-right) using different level of palindromic content as cutoff for 60-mer candidate probes on pig chromosome 14 (*Sus Scrofa* Build 10, NC_010456.3) region from 74377028 bp to 78176022 bp, and on the lower panel shows their distributions after filtering with standard probe selection criteria.

For the two uniqueness scores (Figure 5), unlike for the Agilent catalog array, their distributions were far from normal, both having a peak at zero and a flat, uniform interval followed by a narrow, bell-shaped region. This “twin-peak” distribution makes any formal statistical tests infeasible. However, by thresholding on palindromic content, we determined the density of uniqueness scores for candidates with palindromic content higher than the cutoff; in this way, we directly visualized sequences with higher palindromic content which are subject to removal in the probe selection process. When using a higher cutoff the trend of differences resembles that observed for the Agilent chip: the density curves tilt left, with both peaks shrinking and the saddle region raising. In particular, the proportion of sequences with close to 0 uniqueness score remained large in the high-palindrome group (>60%), yet the high uniqueness peak vanished.

Finally, we further filtered out candidates violating those probe selection parameters used in the design, which include a narrow band of melting temperature (69°C≤Tm≤74°C), moderate GC content (30%≤GC≤50%), no base exceeding 60%, and no single and di-nucleotide repeats exceeding the thresholds of 6 and 4 respectively. After filtering, a similar pattern persists, yet is not so pronounced, suggesting that the filtering removed more candidates with low uniqueness scores. One particular feature is that the candidates with high palindrome (>60%) and 0 uniqueness scores were removed by filtering.

### Benchmarking of public array data

We further used our penalized uniqueness score to evaluate array data from public repository. We choose one popular platform from Agilent, human whole-genome expression array 4x44K (ArrayExpress: A-AGIL-28), which contains 41000 unique probes. We randomly selected from this platform 14 single color array datasets all having proper replicate (at least 2) for each factor level. Probes are scored using our penalized uniqueness score. For each experiment, like running a practical microarray analysis we performed pre-processing and filtering, details could be found in method. In the end, we derived summary statistics of the penalized uniqueness score for filtered probes (Table 4), which are over-saturated or outlying and thus could be related to cross-hybridization. For the analyzed experiments, the number of probe filtered varies from 1 to 458. Interestingly, for all except one experiment (E-GEOD-35635), those filtered probes exhibit lower average *U*_*p*_ score and thus are less unique according to our measurement. To achieve statistical power of 0.9 when using a two sided *t*-test to detect a mean difference of 0.5 with a standard deviation of 1 would require at least 85 observations in each sample. Therefore, we only perform *t*-test for those experiments which has more than 85 probes filtered out. In all three experiments significantly lower uniqueness scores are detected for those filtered probes.

**Table 4:** ArrayExpress experiments summary and penalized uniqueness score comparison

**E-GEOD-ID**	**Array no.**	**Factor level**	**Filtered probe no.**	**Filtered probe mean/median**	**Filtered probe std. dev.**	**Normal probe mean/median**	***t *-testp-value**
22072	5	2	18	9.306/8.798	3.235	9.131/10.04	NA
23131	31	3	61	8.735/9.796	3.492	9.132/10.04	NA
23558	32	2	136	4.784/3.503	4.246	9.146/10.05	2.2E-16
23697	70	2	1	0/0	NA	9.131/10.04	NA
24536	52	5	11	7.508/9.957	4.973	9.132/10.04	NA
25623	32	3	1	4.655/4.655	NA	9.131/10.04	NA
27915	20	5	6	9.633/9.899	2.824	9.131/10.04	NA
29288	132	9	9	8.641/9.957	3.965	9.131/10.04	NA
32155	21	7	23	8.48/9.613	3.642	9.132/10.04	NA
32988	48	10	145	6.618/8.636	4.383	9.14/10.04	1.34E-10
33264	49	16	31	9.428/9.957	2.76	9.131/10.04	NA
35635	57	3	1	12.79/12.79	NA	9.131/10.04	NA
35756	32	8	458	4.229/3.396	3.876	9.187/10.04	2.2E-16
37827	87	29	32	9.078/9.701	2.837	9.131/10.04	NA

## Discussion

The probe selection algorithm implemented in this study utilizes the proposed penalized uniqueness score as a controlling criterion of cross-hybridization, together with several other parameters for the flexible tweaking of the positional distribution, optimal hybridization efficiency, and essential constraints of sequence complexity. Profiling Agilent’s catalog probes confirms the appropriateness of our parameter settings. The intention of the algorithm is to allow a part of the probe to overlap with its neighboring tiles, giving higher coverage and resolution for experiments, like a targeted sequencing library preparation. However, it can also be used to design CHIP-chip experiments, which require distantly-spaced probes across large genomic regions. To achieve this, the selection algorithm behaves slightly differently when a negative overlap is specified, and it will not attempt to shift to the 5', if that would induce overlapping. Studies have shown that sequence polymorphisms may affect probe hybridization efficiency [38-40]. Thus common SNPs defined in databases, like dbSNP, could be excluded in the input FASTA files by cutting the region into two new regions, or SNP could be masked with lowercase letters and controlled like repetitive regions.

The design is done on a per-sequence basis thus the memory requirement of the implementation depends on the largest sequence contig in question. For each sequence header defined in the source FASTA files, to calculate the uniqueness score all corresponding MUP entries in the prefix file have to be imported and processed in RAM; each entry will need two integers for the position and the length of the prefix, respectively, to be stored in the array. This in turn suggests that for large genome tiling design - e.g., when covering a whole mammalian chromosome, which could have a length of 3×10^8^ bp, - storing only the prefix array will take about 9 GB of memory on a 64-bit machine. Additionally, the algorithm runs in linear time with various parameters affecting the exact time expectation. Yet further parallelization is easily available either on a per-sequence basis given sufficient memory or on a per-chunk basis, since, in our implementation, the individual sequence is initially pieced into segments with continuous non-ambiguous bases and sequentially processed. Simple per-chunk test utilizing 4 parallel processes further reduced the running time to approximately 45 min for the same designing task that is presented in the comparison section.

Compared to traditional BLAST-like alignment methods, our definition of the penalized uniqueness score makes less parametric model assumptions for the homologous estimation, letting it be more sequence-driven and less sensitive to arbitrary parameter settings. The calculation of the score makes usage of GenomeTools (http://genometools.org/), which is memory relaxed, and inherently benefit from the computational efficiency of the FM-index [41]. As a rational variable, it provides a more continuous distribution and a wider dynamic range for the uniqueness measurement without further increase in the computational complexity, while showing higher sensitivity and specificity over the original count score, which only takes discrete integers ranging from 0 to the user specified maximal length of MUP. The score itself, like the original count score, measures the degree of uniqueness and dissimilarity of the sequence to the rest of the genome, which means the lower the score the higher possibility for non-specific binding. Thus, it could be further factored in the background correction model or at the normalization step prior to the downstream array data analysis, to correct for cross-hybridization noises using the uniqueness score. Such a correction model could take the typical form as the exponential-normal convolution model in RMA algorithm [42,43], by adding an additional term for non-specific binding noise.

Concerning palindrome sequences, which play an important structural role in the biogenesis of microRNA [44-46] and also have other functional characteristics like acting as restriction enzyme sites [47], our results suggest that with higher palindromic content the sequence tends to have a lower uniqueness score, contrary to what has been previously claimed in [25]. Aside from nucleotide sequence, Sheari *et al.* studied the palindrome in protein sequence using a linguistic measurement, in which they also related palindromes with low sequence complexity [48]. Under our uniqueness measurement, lower complexity normally leads to fewer and longer MUS in the region, resulting in a lower penalized uniqueness score. Our experimental observations could also be explained in plain theory, since a highly palindromic sequence will share a large identical segment with its reverse complement strand; thus there should be fewer unique substrings found in such regions.

## Conclusions

In this study, we defined the penalized uniqueness score, which could serve as a better measurement for sequence heterogeneity, showing higher sensitivity and specificity. The efficient and flexible tiling probe selection algorithm we implemented could assist in the design of various types and scales of genome tiling task, with high coverage and resolution, while giving more control of the expected hybridization efficiency. Using Agilent's catalog array and experimental data, our *in silico* analysis revealed that palindromic sequence exhibiting relatively lower uniqueness (measured by uniqueness scores). Re-analyzing public array data further provided support of using the penalized uniqueness score to discriminate non-specific probes for microarray data analysis.

## Methods

### Validation data and method

We fetched the Agilent catalog array Human Whole Genome ChIP-on-Chip Set 244K (1 to 25, available through Agilent’s eArray), which in total contains 5930500 probes spanning the whole human genome (hg19:GRCh37). All probes were processed for the two uniqueness scores, and BLAT was used to find hybridization-quality alignments, which is defined as having at most one gap, at least 60% identity of the probe length, and gap length or mismatched bases not more than 3. Such alignments are considered to be hybridized well, thus the number of qualified alignment could be used as an indicator for cross hybridization potential. According to the chromosomal coordinates provided in the GEO files shipped together, probes were back-mapped to the same version of RepeatMasker-masked genome sequence (hg19:GRCh37). Proportion of masked bases was calculated for each mapped probe.

### Receiver operating characteristic curve

To access directly the relative performance of the two scores, receiver operating characteristic curve (ROC) was adopted measuring the trade-off between sensitivity and specificity. To construct the curve, we used a series of values ranging from the minimum to the maximum of the uniqueness score as cutoffs to predict whether the probe has only one hybridization-quality alignment or more. If the probe has a uniqueness score higher than the cutoff, then it is classified as positive and estimated to have only one quality alignment, and negative otherwise. Sensitivity is defined as the true positive rate, which is the number of probes identified as positive and indeed having only one quality alignment divided by the number of probes with an alignment score equaled to one. Specificity is defined as 1 minus the false positive rate, which is the number of probes identified as positive but having more than one quality alignment divided by the number of probes actually having one quality alignment.

### Design comparison setup

To address the coverage and resolution of our design, we chose human chromosome 22 (GenBank:NC_000022.10) as the target region, and the reference genome sequence was downloaded from the NCBI archive. Knowing that, for experiments like CHIP-chip, the sheared chromatin fragment is generally around 500bp [49], using short oligonucleotides (< 100-mer) makes it more cost-efficient to choose an optimal distance between tiles than to solely increase the number of probes and the tiling density. To compare with Agilent's catalog design, we analyzed the distribution of the distance between neighboring tiles in the catalog array, and we found a median of 202 nt and mean of 426 nt. The probe length also varied, ranging from 45-mer to 60-mer (median 53; mean 52.85). With our implementation, we tested several overlapping sizes (from −325 nt to −250 nt) to make the overall probe number close to the Agilent catalog design. The minimal probe length was set to 45-mer, while initial probe length always starts at 60-mer. For Tm, in [25] they used the simple GC model [50] and tested two ranges of temperatures, 73–76°C and 77–80°C, which in turn correspond to a GC content range of around 30-50% for a 50-mer oligo. In contrast, by considering the Tm distribution in the Agilent catalog array (see Additional file 1), we empirically selected a Tm range of 69–74°C for the nearest neighbor model and combined it with a controlled GC content range of 30-50% as our criteria for optimal hybridization efficiency. For cross-hybridization control, we used a penalized uniqueness score of 9 as the empirical cutoff. A threshold of 30% for palindrome was used, while all other parameters were kept as the following: no base proportion higher than 60%, single nucleotide repeats not exceeding 6 and not having more than 4 di-nucleotide repeats.

### Pre-processing and filtering of public array data

For the preprocessing and filtering of the ArrayExpress datasets, we used Bioconductor [51] package Agi4x44PreProcess. Experimental designs were derived according to individual experiment description. Procedural and parametric settings of background-correction, normalization, and filtering were set to default and common to all experiments. The filtering in this package is done sequentially: first, control probes are filtered; then probes with signal not well above the local background are filtered; the third criterion is by the Agilent's FeatureExtraction flag 'gIsFound'; for the next step, probes with signal not well above the negative controls are filtered. So far, the remaining probes all have detectable and valid signal. In the next stage, over-saturated probes are removed, which could be linked to severe cross-hybridization or possible contamination to the slide; In the end, population outliers and non-uniform outliers are filtered, which could also be related to cross-hybridization or other variations in experimental conditions. In this work, we considered probes filtered out in the last 2 stages as potential victims of cross-hybridization and had them further investigated for uniqueness.

### Data analysis

The tiling probe selection algorithm and penalized uniqueness score computation were implemented as a Perl program. MUP was calculated using GenomeTools (http://genometools.org/). All analysis and visualization were performed using R [52] unless otherwise stated.

## Availability and requirements

Project name: OTAD

Project home page:

http://www1.fbn-dummerstorf.de/en/forschung/fbs/fb3/paper/2012-yang-1/OTAD.v1.1.tar.gz

Operating system(s): Linux

Programming language: Perl

License: GNU GPL (see separate license for genometools).

## Competing interests

The authors’ declare that they have no competing interests.

## Authors’ contributions

YD developed methodology, implemented the algorithm, performed data analysis and wrote the manuscript. EM and SK provided the data and critically revised the manuscript. KW conceived this study and critically revised the manuscript. All authors contributed to the discussions for the improvement of the original draft and approved the final manuscript.

## Supplementary Material

Additional file 1 **Supplement.** Design parameters distribution of Agilent ChIP-on-Chip set probes. Click here for file
