# Supplementary material for: Flexible and efficient genome tiling design with penalized uniqueness score
Source: BMC Bioinformatics. 2012 Dec 5;13:323. doi: 10.1186/1471-2105-13-323 (PMC3583072; doi:10.1186/1471-2105-13-323)
Supplement: Additional file 1 — Supplement. Design parameters distribution of Agilent ChIP-on-Chip set probes. [file 1471-2105-13-323-S1.pdf]

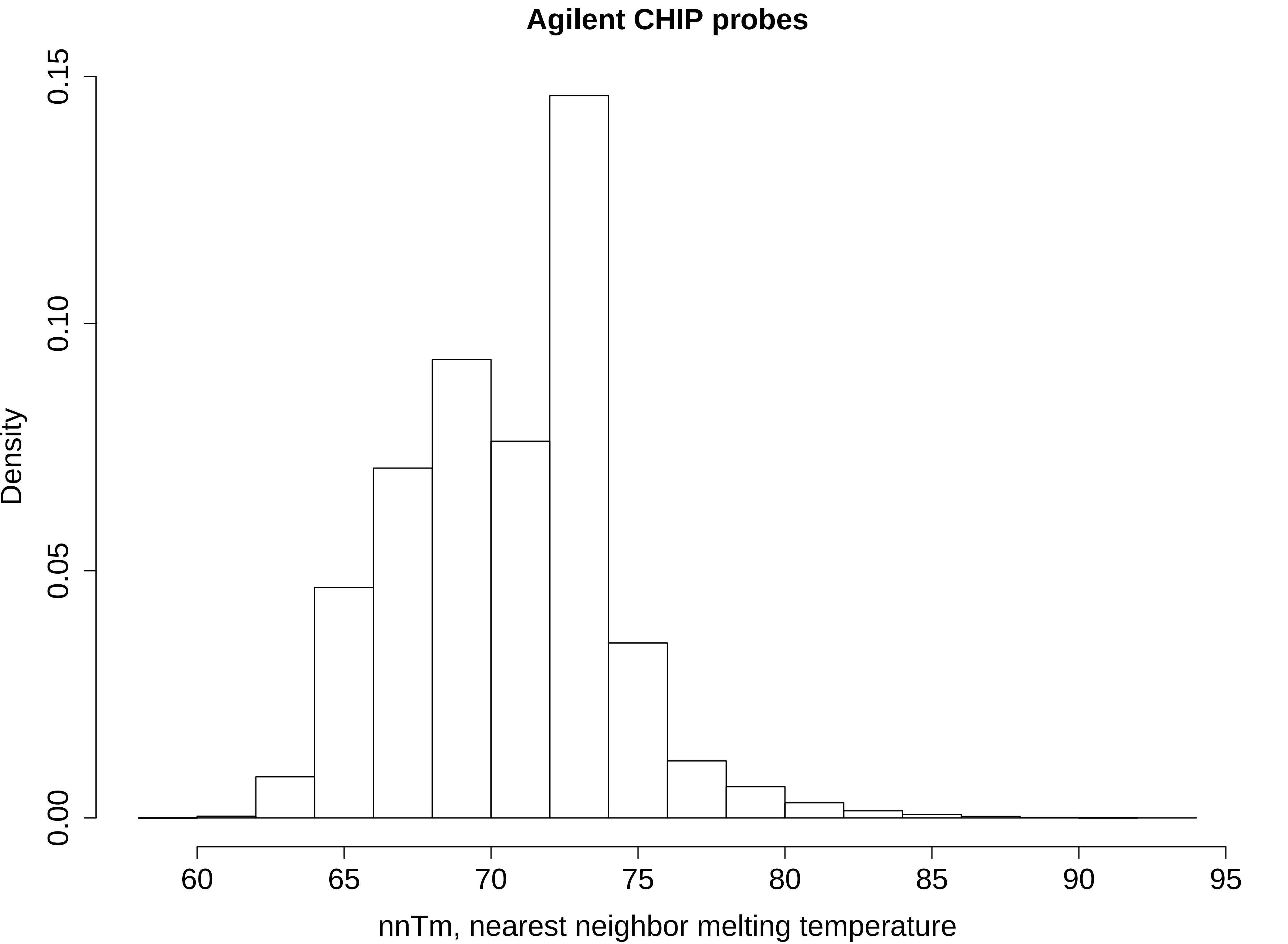
Agilent CHIP probes
Density
0.00
0.05
0.10
0.15
60
65
70
75
80
85
90
95
nnTm, nearest neighbor melting temperature

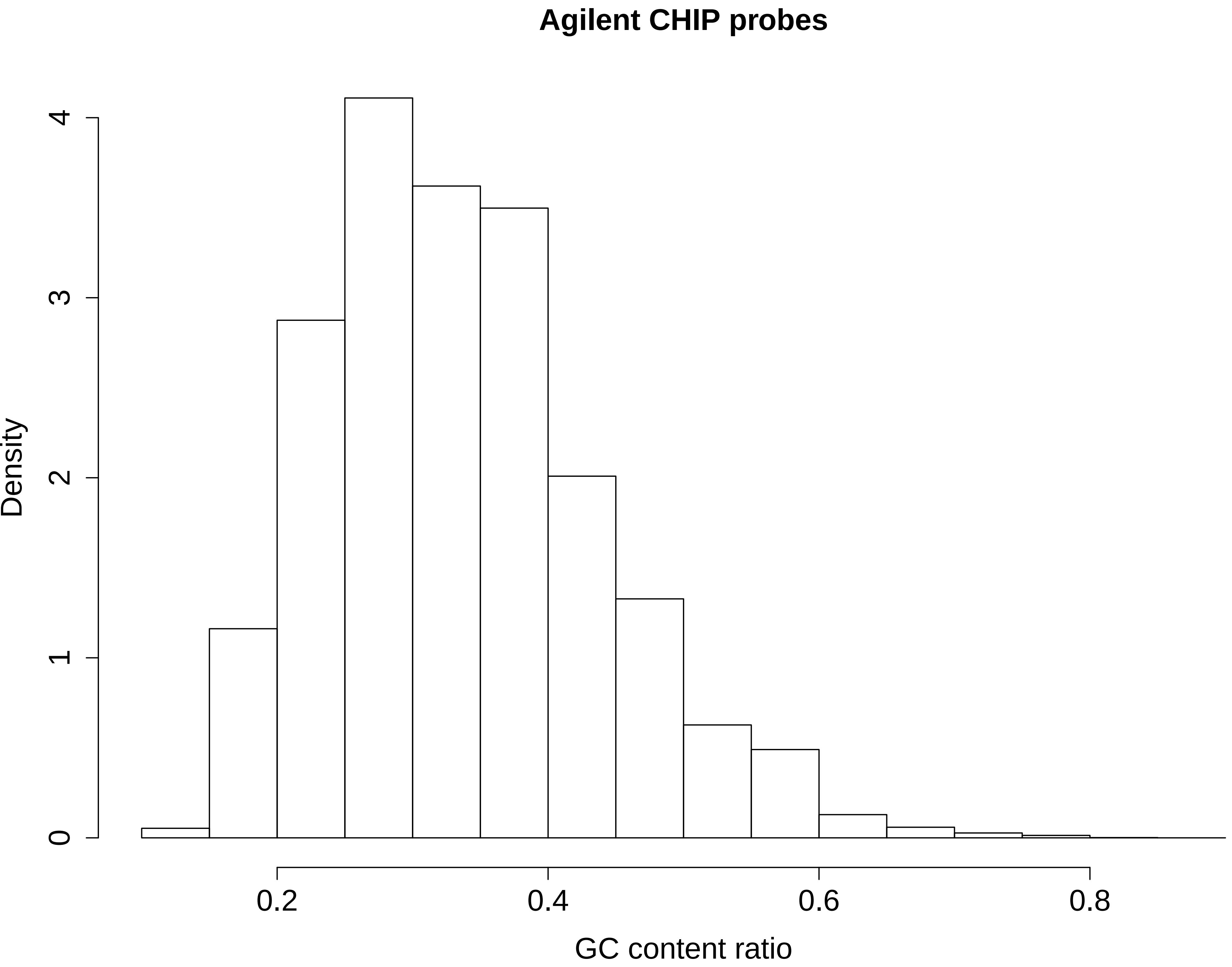
Agilent CHIP probes
Density
0
1
2
3
4
0.2
0.4
0.6
0.8
GC content ratio

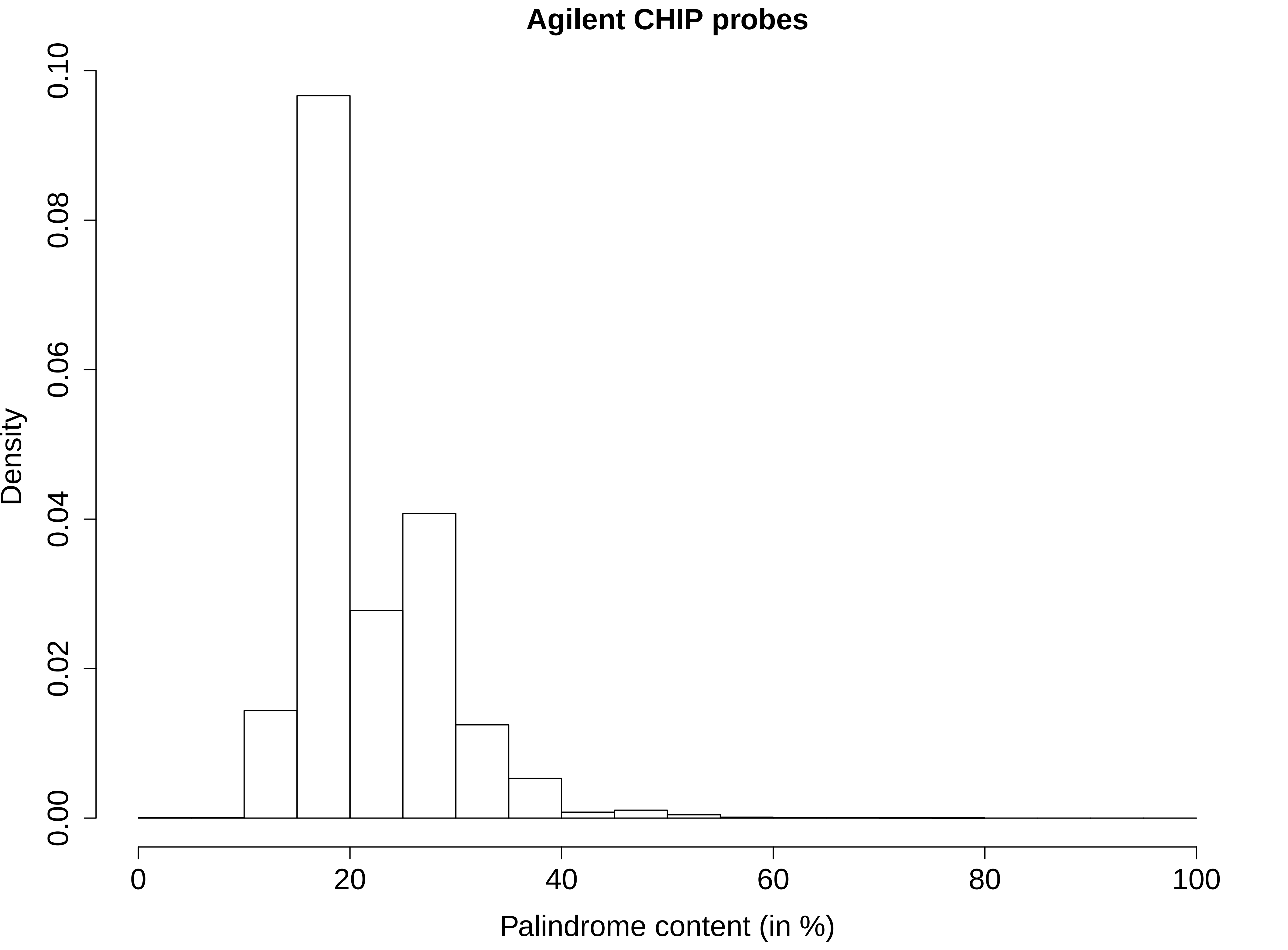

Agilent CHIP probes
Density
0.00
0.02
0.04
0.06
0.08
0.10
0
20
40
60
80
100
Palindrome content (in %)

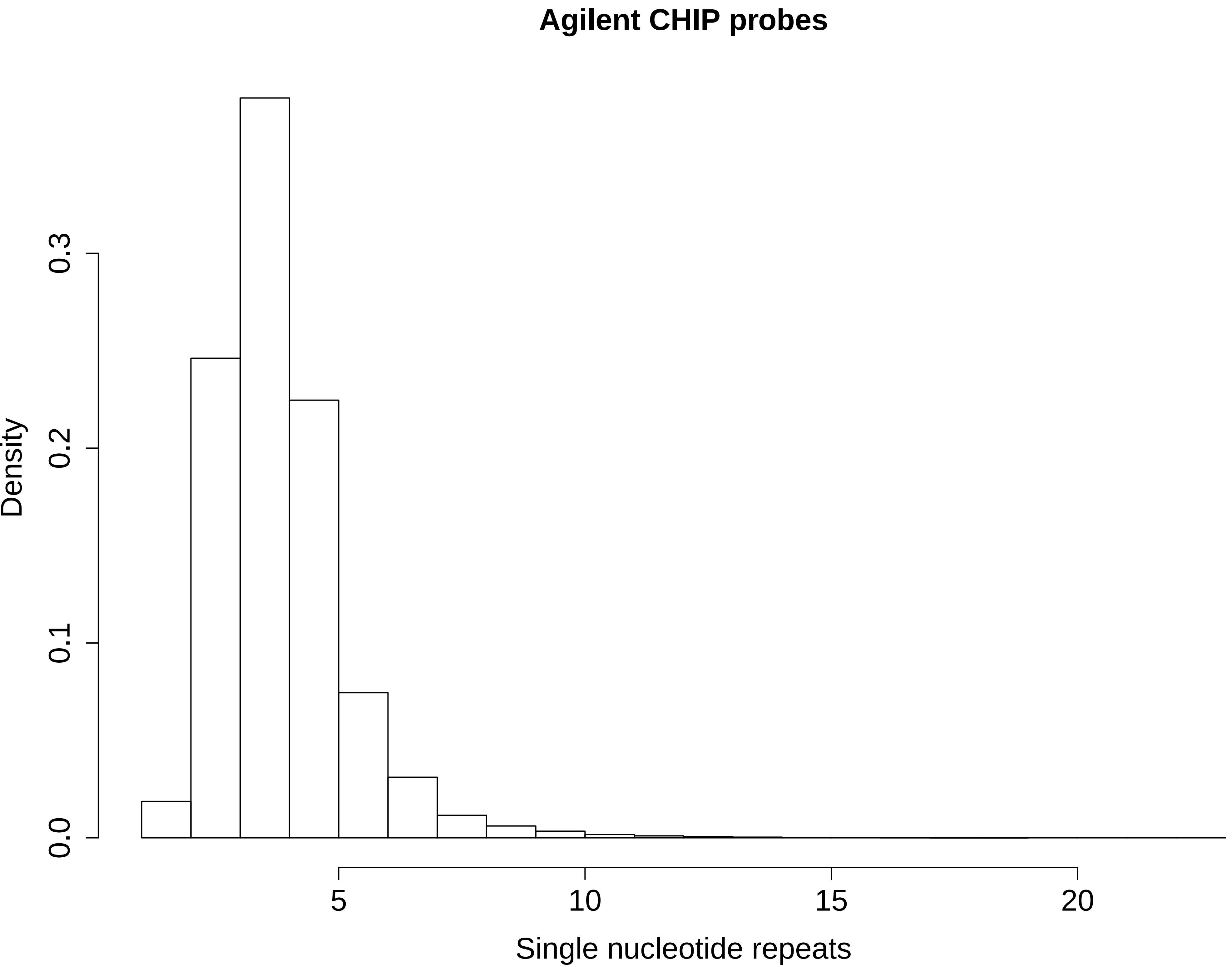

Agilent CHIP probes
Density
0.0
0.1
0.2
0.3
5
10
15
20
Single nucleotide repeats

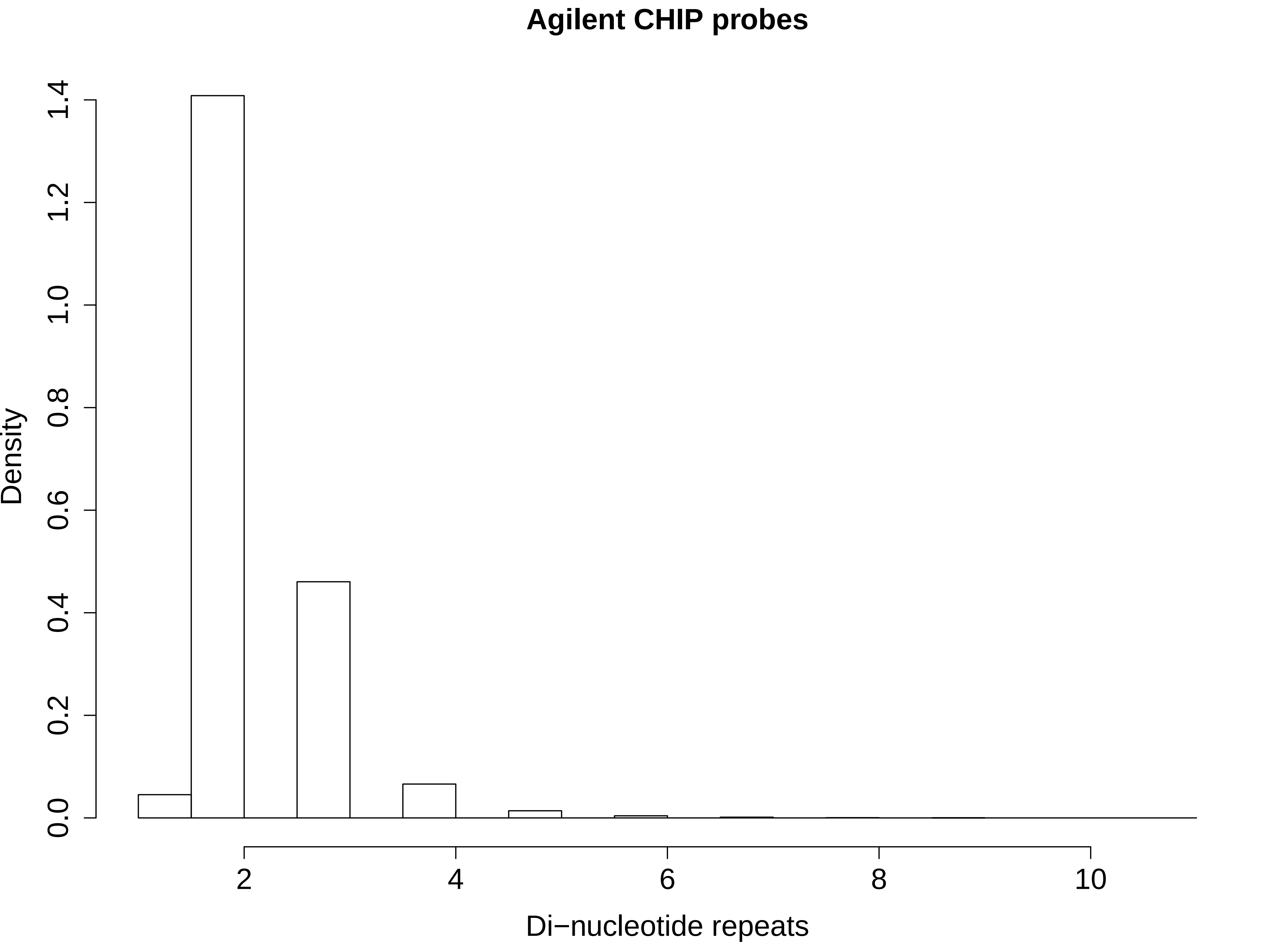

Agilent CHIP probes
Density
0.0
0.2
0.4
0.6
0.8
1.0
1.2
1.4
2
4
6
8
10
Di−nucleotide repeats

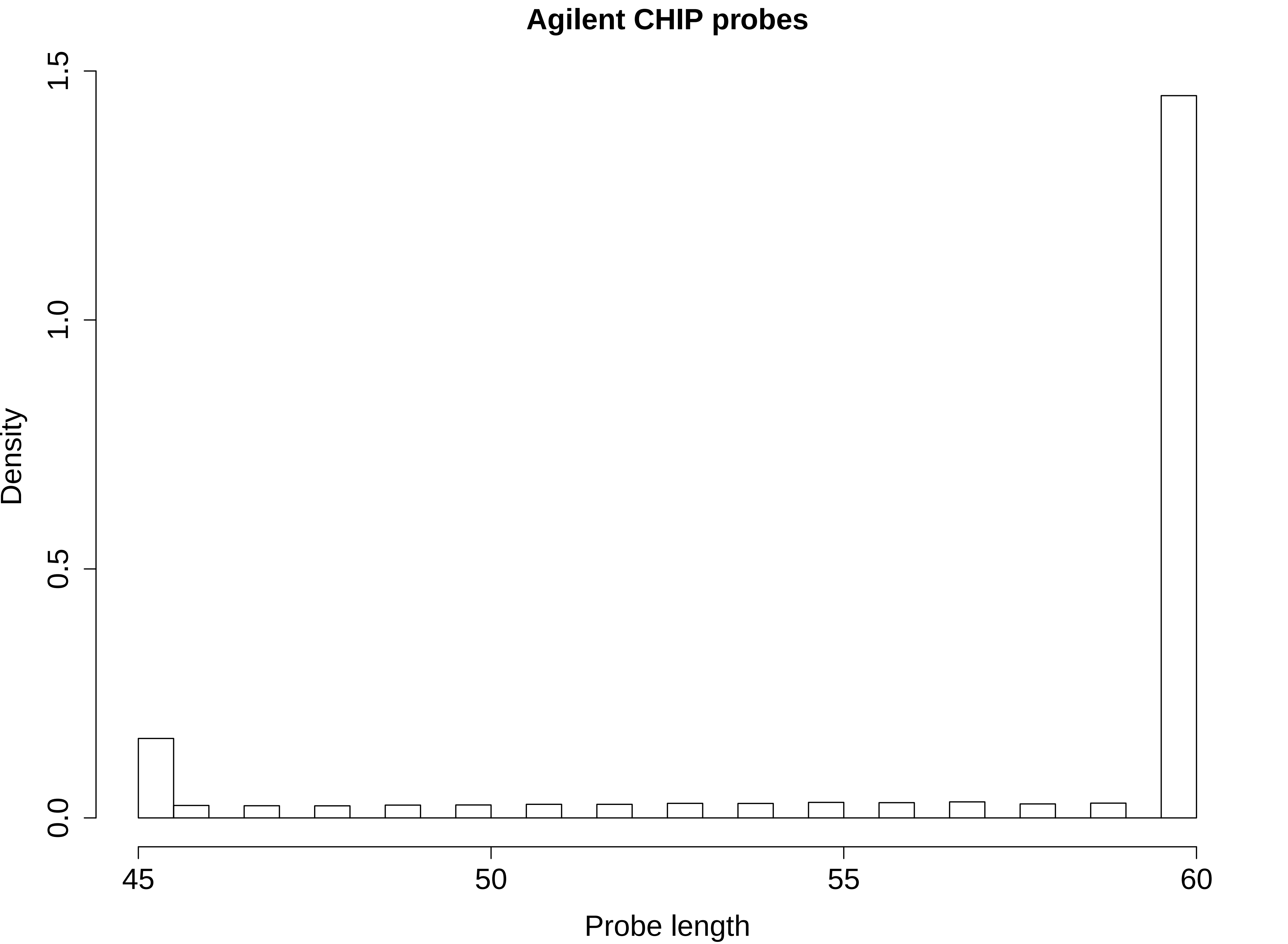

Agilent CHIP probes
Density
0.0
0.5
1.0
1.5
45
50
55
60
Probe length

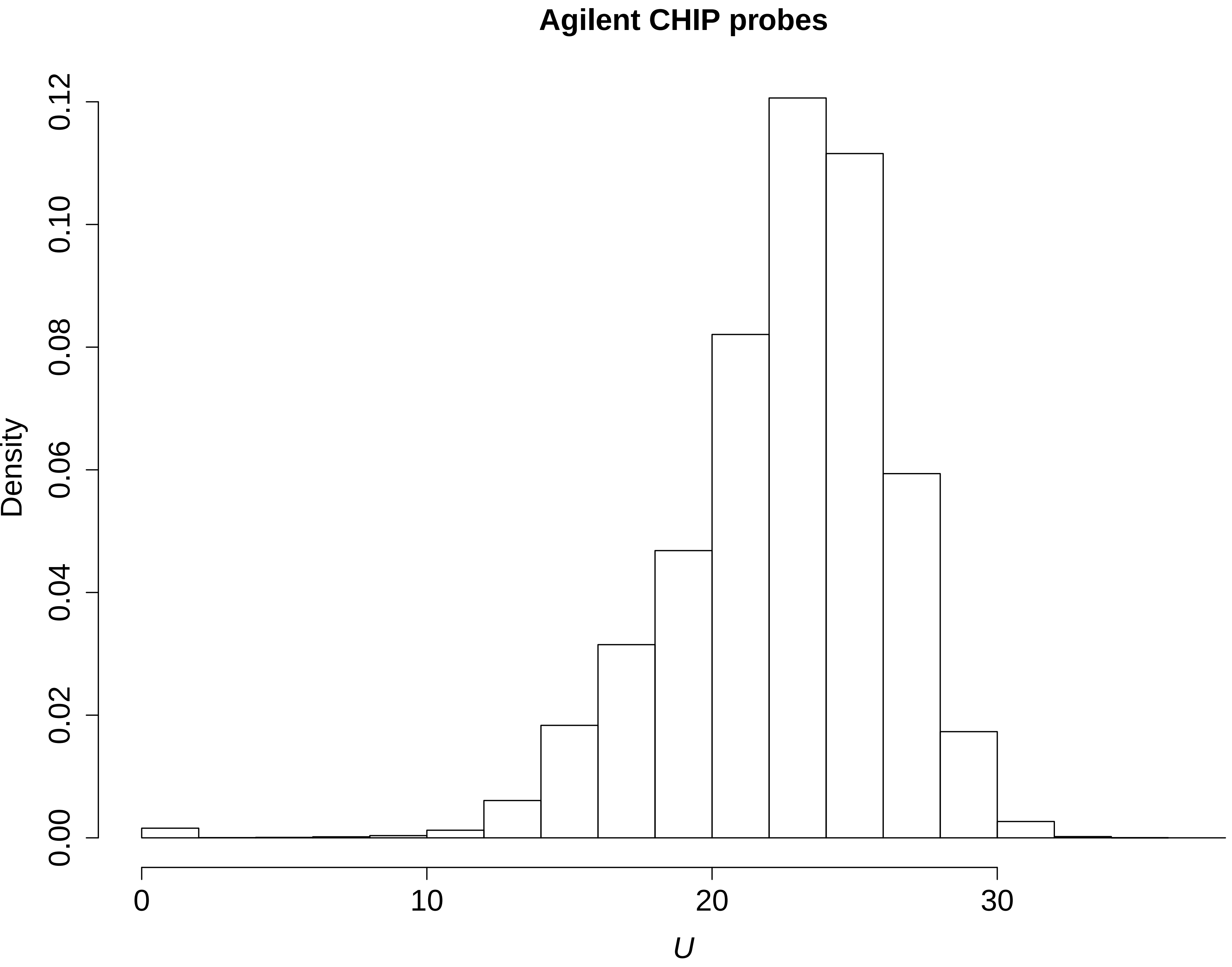

Agilent CHIP probes
Density
0.00
0.02
0.04
0.06
0.08
0.10
0.12
0
10
20
30
U

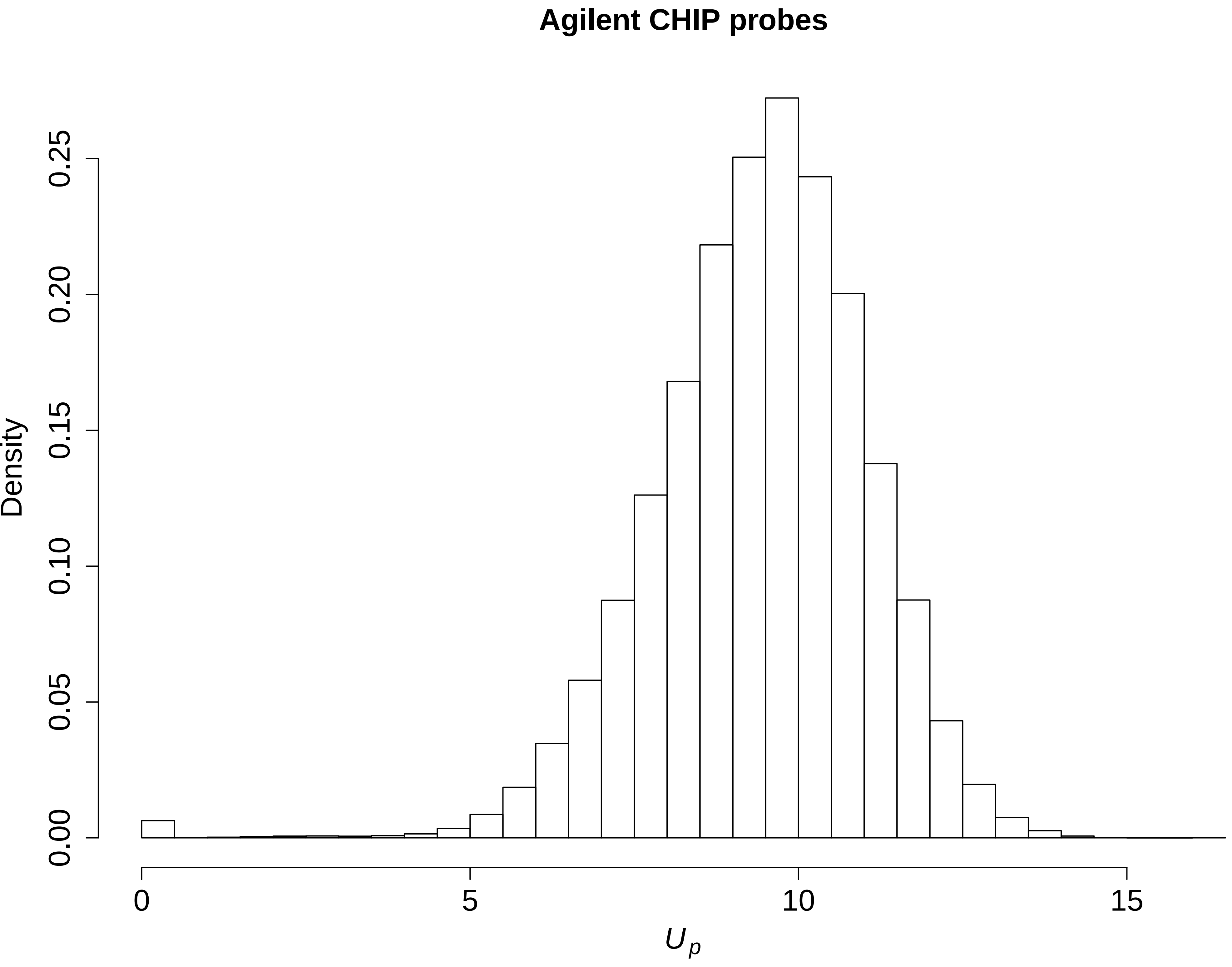

Agilent CHIP probes
Density
0.00
0.05
0.10
0.15
0.20
0.25
0
5
10
15
U_p
